# Tissue Engineering to Improve Immature Testicular Tissue and Cell Transplantation Outcomes: One Step Closer to Fertility Restoration for Prepubertal Boys Exposed to Gonadotoxic Treatments

**DOI:** 10.3390/ijms19010286

**Published:** 2018-01-18

**Authors:** Federico Del Vento, Maxime Vermeulen, Francesca de Michele, Maria Grazia Giudice, Jonathan Poels, Anne des Rieux, Christine Wyns

**Affiliations:** 1 Gynecology-Andrology Unit, Medical School, Institut de Recherche Expérimentale et Clinique, Université Catholique de Louvain, 1200 Brussels, Belgium; federico.delvento@uclouvain.be (F.D.V.); vermeulen.maxime@live.be (M.V.); francesca.demichele@uclouvain.be (F.d.M.); giudicemariagrazia@gmail.com (M.G.G.); 2 Department of Gynecology-Andrology, Cliniques Universitaires Saint-Luc, 1200 Brussels, Belgium; jonathan.poels@uclouvain.be; 3 Advanced Drug Delivery and Biomaterials Unit, Louvain Drug Research Institute, Université Catholique de Louvain, 1200 Brussels, Belgium; anne.desrieux@uclouvain.be

**Keywords:** prepubertal, male fertility, fertility preservation, fertility after cancer, spermatogenesis, testicular tissue, spermatogonial stem cells, transplantation, tissue engineering, nanoparticles

## Abstract

Despite their important contribution to the cure of both oncological and benign diseases, gonadotoxic therapies present the risk of a severe impairment of fertility. Sperm cryopreservation is not an option to preserve prepubertal boys’ reproductive potential, as their seminiferous tubules only contain spermatogonial stem cells (as diploid precursors of spermatozoa). Cryobanking of human immature testicular tissue (ITT) prior to gonadotoxic therapies is an accepted practice. Evaluation of cryopreserved ITT using xenotransplantation in nude mice showed the survival of a limited proportion of spermatogonia and their ability to proliferate and initiate differentiation. However, complete spermatogenesis could not be achieved in the mouse model. Loss of germ cells after ITT grafting points to the need to optimize the transplantation technique. Tissue engineering, a new branch of science that aims at improving cellular environment using scaffolds and molecules administration, might be an approach for further progress. In this review, after summarizing the lessons learned from human prepubertal testicular germ cells or tissue xenotransplantation experiments, we will focus on the benefits that might be gathered using bioengineering techniques to enhance transplantation outcomes by optimizing early tissue graft revascularization, protecting cells from toxic insults linked to ischemic injury and exploring strategies to promote cellular differentiation.

## 1. Introduction

While oncological treatments can cure more than 80% of pediatric cancers in Europe [1], chemotherapeutic agents and radiotherapy have deleterious effects on the gonads of prepubertal boys [2,3]. Moreover, the risk of permanent infertility is also high when preconditioning therapies are applied before bone marrow transplantation to cure benign conditions such as hemoglobinopathies [4].

Loss of fertility significantly compromises quality of life [5], and surveys performed in cancer survivors have shown that 80% of these patients are considering parenthood, although only 10% of them would use donor sperm or choose adoption [6,7]. Between 2005 and 2013 in our institution, the acceptance rates of the fertility preservation procedure justified by an oncological diagnosis were 74% for boys under 12 years and 78.6% for boys aged 12 to 18 years [7]. Similar rates were reported in USA centers [8] and are expected to be alike in Europe based on a questionnaire offered to child cancer survivors’ parents [9]. This illustrates the significant importance of fertility for minor patients and their parents and fully justifies the development of fertility preservation strategies in pediatric populations.

If, for post-pubertal patients, sperm cryopreservation is the gold standard for fertility preservation, for young patients who do not yet produce mature gametes, cryopreservation of immature testicular tissue (ITT) (containing spermatogonial stem cells (SSCs), which are spermatozoa precursors [10]) or germ cells suspension can be offered before gonadotoxic therapies to preserve their fertility [3].

A controlled slow freezing procedure using dimethyl sulfoxide as the main cryoprotectant is commonly applied to cryopreserve small ITT fragments (2–4 mm^3^) taken from one testis, with sampling limited to less than 5% of the testicular volume [11,12,13,14]. Using a xenotransplantation assay in nude mice to evaluate the cryopreservation procedure, the integrity of the SSC niche that regulates stem cell self-renewal and differentiation [15] appeared to be well preserved [16].

**Figure 1:**
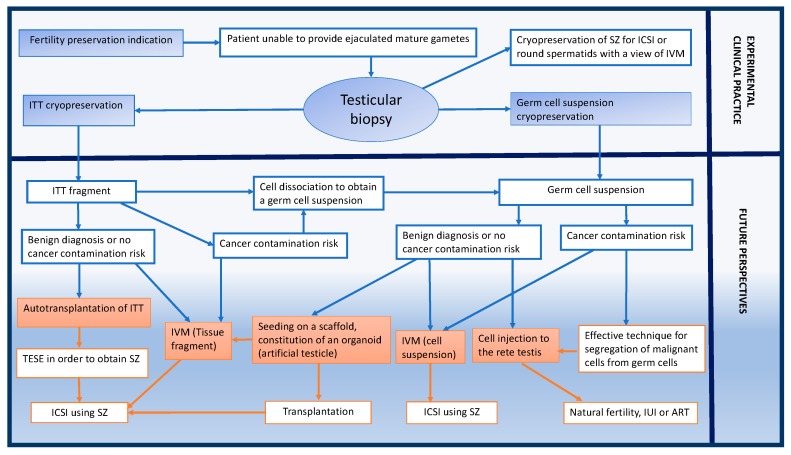
Fertility preservation for peri- and pre-pubertal male patients: experimental clinical practice and future perspectives. SZ: Spermatozoa; ICSI: Intra-Cytoplasmic Sperm Injection; ART: Assisted Reproductive Technology; IVM: In Vitro Maturation; TESE: TEsticular Sperm Extraction; IUI: Intra-Uterine Insemination.

Restoring patient’s compromised fertility by obtaining mature gametes production from stored cells or tissue might be achieved through auto-transplantation or in vitro maturation once the cytotoxic treatment is completed (Figure 1).

The choice between different options must be made considering the disease of the patient. Since intratesticular contamination by malignant cells was described in approximately 21% of boys with leukemia [17] and xenotransplantation in nude mice of rat testicular cells contaminated by leukemia cells brought tumor transmission in the host [18], auto-transplantation of frozen-thawed gonadal tissue fragments may only be considered for fertility restoration purposes when there is no risk of retransmission of cancer cells to the cured patient. Such risk must also be evaluated for other blood malignancies and for metastasizing cancers. Selection of SSCs before transplantation may therefore be an alternative for these patients if proof can be made that the cell sorting technique using post-purification examination of the prepared samples is safe [19].

Otherwise, in vitro maturation (IVM) of human prepubertal spermatogonia [20], which is currently under investigation, will be the only option; however, it will not be addressed in this review.

## 2. Results and Discussion

### 2.1. Lessons Learned from Transplantation of Immature Testicular Tissue Fragments

While fertility restoration with transplantation of cryopreserved human ITT is still at a preclinical stage, autotransplantation of frozen-thawed ovarian cortex [21] in the context of fertility preservation has already proven clinically efficient [22]. Over 100 live births have been reported worldwide whether using adult [23] or prepubertal tissue [24]. However, further progress is still awaited considering the important loss of ovarian follicles following transplantation assays in mice due to hypoxia/reoxygenation injuries [25] and to a massive primordial follicle activation [26]. Different strategies, based on tissue engineering approaches, have been tested to limit these phenomena, like follicle encapsulation in fibrin hydrogel and vascular growth factor administration [23].

With regard to transplantation of cryopreserved testicular tissue, grafting of human ITT has been so far exclusively performed in host nude mice [12,16,27,28,29,30,31]. A summary of human prepubertal and postpubertal testicular tissue grafting experiments is provided in Table 1.

Poor graft survival [32] and massive germ cells loss [27] were observed especially after mature testicular tissue ectopic xenotransplantation. After orthotopic ITT xenotransplantation using the same nude mouse model, there seemed to be a worsening of spermatogonial survival rates over time (61% at 5 days, 14.5% at 3 weeks, and 3.7% at 6 months [12,16,31]). Such reduction in germ cell numbers might likely be the result of tissue degeneration caused by the hypoxia that occurs before the generation of an early blood supply, as was observed in ovarian transplantation experiments [25]. Moreover, further germ cell loss continues with the highest proportion of the loss happening within the first three weeks, suggesting the importance of a stable vascularization for an efficient grafting technique [33]. Interestingly, as spermatogonial recovery rates decrease, seminiferous tubules’ structures partially recover over time (18–21% at 5 days, 82.2 ± 16.5% at three weeks, 89.7 ± 17.9% at 6 months), although presenting more niches that do not contain spermatogonia [12,16,30,31]. As qualitative analysis of Leydig cells showed a good preservation at the protein and ultrastructural levels with maintenance of their steroidogenic potential [16], and as Sertoli cells proliferation was found to be superior to spermatogonial proliferative activity three weeks after xenografting [12,31], it seems that the germ cell population is mainly affected. It has thus been postulated that the transplantation procedure and the recipient environment could be responsible for an increased recruitment of SSCs entering the differentiation process, thus reducing the number of SSCs capable of auto-renewal [31].

Furthermore, while the survival of proliferating spermatogonia was observed, their differentiation potential was limited to the primary pachytene spermatocyte stage, although spermatid-like and spermatozoon-like structures were described using standard and transmission electron microscopy [16]. The phylogenetic distance between species was suggested to be responsible for the abnormal differentiation, but this should be further confirmed after the development of a validated model able to assess tissue and cell functionality including its maturation (see Section 2.11).

Moreover, the pachytene spermatocyte stage was reached within 6 months after orthotopic xenotransplantation in castrated nude mice of tissue obtained from boys under the age of 10 years [16,30]. However, the endocrine exposure needed for the physiological pubertal transition period and onset of spermatogenesis in boys may last between two and four years [36], which may also be a factor that could influence the maturation stage achieved after 6 months xenotransplantation.

By contrast, in other species, complete spermatogenesis was reported after both autologous transplantation [37,38,39] and xenotransplantation [37,39,40,41,42,43] and even led to offspring in some species (including non-human primates) after prepubertal testicular tissue autotransplantation [39,44] and xenotransplantation [39,45,46,47]; for a complete review, see [48].

Besides the phylogenetic distance between species and the length of the pubertal transition period, a number of other parameters such as the avascular grafting procedure, the grafting site [12], the maturational stage of donor’s tissue [16,49], and other donors’ characteristics [50] may have an impact on graft development and functionality.

#### 2.1.1. Transplantation Technique

In 2002, Honaramooz et al. grafted on the back skin of nude mice fresh small ITT biopsies retrieved form prepubertal mice, pigs, and goats obtaining a complete spermatogenesis with all three species’ tissue. According to the report, ITT fragments were simply fixated subcutaneously without the constitution of an artificial vascular anastomosis [37]. A vascular connection between the grafted tissue and the host was then spontaneously established, with vessels outgrowing from the transplanted fragment and then connecting to the host’s vascular system [51]. As the cell-to-capillary distance is limited to 200 μm, survival of any tissue exceeding this dimension requires a mature and stable vascularization [52]. Therefore, even very small testicular tissue biopsies (±1 mm³) are exposed to a period without blood supply characterized by hypoxia, followed by a phenomenon of reoxygenation as already demonstrated for ovarian tissue grafting [25].

#### 2.1.2. Transplantation Site

Transplantation of rodent testicular tissue has been performed in several sites, such as the anterior chamber of the eye [53], the back skin [37], the testis [39], and the ear tip [54], with results in terms of germ cells differentiation that suggested a negative impact of higher temperatures on grafts, hence pointing out orthotopic grafting as the best option [12].

Marmoset ITT autologous ectopic transplantation encountered a differentiation blockade at the spermatocyte stage [55], although interestingly, complete spermatogenesis was detected after autologous orthotopic transplantation in prepubertal castrated host [56].

Intra-testicular grafting has also been performed, and according to the authors of these reports, removing testicular parenchyma could create rupture of seminiferous tubules continuity, facilitating recolonization by SSCs from the donor. Therefore, intra-testicular graft (though technically more complicated) has been proposed as a useful alternative option for intra-scrotal ITT grafting [57].

#### 2.1.3. Maturational Stage of Donor Tissue

The possible impact of the age of the donor on testicular tissue graft outcome has been postulated in many reports on animal experiments [49,58,59]. In agreement with these observations, a higher degree of maturity of human prepubertal tissue also appeared to play a role, as reported by Wyns et al. in 2008. The authors observed a very low recovery rate in the two 14-year-old patients, where focal spermatogenesis was already present in fresh tissue, compared to the better results obtained with younger (12 and 7 years old) donor tissue after xenografting [16].

#### 2.1.4. The Host Environment

In addition to all these non-modifiable characteristics, the nude mouse model itself might be responsible for the germ cell maturation arrest encountered after ITT xenotransplantation. Experiments with non-human primate tissue provided useful and challenging information. Rhesus monkeys’ ITT led to complete spermatogenesis after autologous transplantation [38] but not after xenotransplantation in nude mice [60], proving host environment to be critical for spermatogenic differentiation.

These results suggest that differences in gonadotrophins structure and the endocrine environment could be involved in graft’s germ cell differentiation.

Several strategies have been proposed to reach further progress using available study models, such as acting on the endocrine environment or sheltering spermatogonia from hypoxic insults either by reducing the ischemic period before revascularization or by using protective molecules. These reports not only pioneered in ways to improve the transplantation techniques, but they also provided insight and information on the physiology of (xeno)-transplanted testicular tissue.

### 2.2. Acting on the Endocrine Environment

The hypothalamic–pituitary–testis axis manages the induction and maintenance of spermatogenesis [36], as well as the endocrine function of the testicle.

Spermatogonia lie in the seminiferous tubule in strict contact with surrounding cells, in a functional unit called the stem cell niche, and it is the relationship between soluble molecules, cells cohesion within the niche, endocrine feedback, and paracrine environment that modulates spermatogenesis and spermatogonial self-renewal (for review see Potter et al., 2017) [61]. Better results for mouse ITT intra-testicular grafting compared to SSCs rete testis injection were ascribed to the preservation of the spermatogonial paracrine microenvironment, which allows spermatogenesis to be supported by the original Sertoli cells [12].

During xenografting experiments castrated hosts have frequently been used. After bilateral orchiectomy, the absence of testosterone production causes an interruption of the relationship between the testicle and the pituitary gland, leading to increased gonadotrophin levels above those present at physiological pubertal onset [62]. This affects testicular maturation and might contribute to the acceleration of the differentiation process of the immature tissue previously reported in non-human primates and human tissue [28,63].

The restoration of a functional endocrine feedback between the grafted tissue and the host hypothalamic-pituitary-gonadal axis has been proven. Indeed, 4 weeks after allotransplantation of prepubertal mouse tissue in castrated mice, a decrease of FSH levels to precastration values was observed [62], and grafted ITT supplied normal to elevated levels of androgens to the castrated donors [37]. This was further corroborated by increased weights of seminal vesicles in grafted hosts [37]. However, mouse hormonal hyper-gonadotropic environment after bilateral orchiectomy has been exonerated from the responsibility of the spermatogenic blockade, as similar results were obtained after human ITT xenografting in non-castrated mice (when intra-testicular transplantation was performed) [29].

Questions have been raised about the actual benefit that external intervention on the endocrine environment might add to the grafting procedure. Indeed, hamster ITT xenografts were proven to be mainly regulated by intrinsic mechanisms, as they grew to an analogous size when grafted to nude mice with different degrees of castration (hemi or complete) [64].

In addition, co-grafting in nude mice of ITT from two species, Hamster and Marmoset, where opposite results after xenografting were obtained (the prior going through complete spermatogenesis and the latter who did not and did not even achieve androgens release) [49] showed that the increase in testosterone supplied by the Hamster tissue was not able to overcome the limitations of xenografting of Marmoset ITT [65]. Furthermore, exogenous hCG also remained inefficacious to achieve spermatogenic differentiation [65].

On the contrary, administration of exogenous gonadotropins to recipient castrated nude mice led to complete spermatogenesis using infant rhesus monkeys ITT xenografts [66,67], and it improved tissue maturation and differentiation after xenografting of ITT from prebubertal horses [68].

Thyroid hormones play a well-known role in spermatogenesis and hemato-testicular-barrier function [69], and interaction between grafted testicular tissue and the recipients’ thyroid was established. Indeed, administration of propylthiouracile, a disruptor of thyroid axis function, reduced graft efficiency and germ cells differentiation in bovine ITT xenografts in nude mice [70].

As far as it concerns human ITT, two reports attempted to artificially modify the recipient mouse endocrine environment to assess its influence on the xenografts. In a first experiment, subcutaneous administration of exogenous recombinant human follicle stimulating hormone (FSH) was performed after human ITT intratesticular xenografting in nude mice [29], and in a second experiment, ITT xenografting in the scrotal bursa of nude mice was followed by intraperitoneal testosterone supplementation with a fixed dose of intramuscular human FSH in order to counterbalance the negative feedback on gonadotropins secretion [31]. None of the graft hormone supplementations promoted spermatogonial differentiation nor restored spermatogenesis, although combined administration of FSH and testosterone improved intra-tubular cellular proliferation of both spermatogonia and Sertoli cells.

The way hormones were administered might have been unsuitable to elicit any desired effect but other molecular mechanisms might be implicated, such as a disrupted cellular response caused by the grafting procedure, or an interaction with the host’s own gonadotropins or with its thyroid-gonadal axis [70].

### 2.3. Reducing Ischemia Due to the Avascular Transplantation Procedure

As germ cells, and more specifically differentiating spermatogonia, are highly vulnerable to the hypoxia insult [71], reducing the ischemic period through enhanced vascularization using vascular growth factors might have beneficial effects on SSC survival.

Vessels formation occurs by a complex dynamic process governed by several pro and anti-angiogenic molecules [72], and several growth factors such as vascular endothelial growth factor (VEGF), platelet derived growth factor (PDGF), and fibroblast derived growth factor (FGF) have been tested for distinct therapeutic applications including tissue regeneration and neovascularization of ischemic tissues, although clinical trials results were mainly disappointing [73,74].

Beyond its role in angiogenesis, VEGF is also involved in the support of germ cell survival and self-renewal [75] through receptors whose expression has been demonstrated on spermatogonia, and Sertoli and Leydig cells [76]. This makes VEGF an excellent candidate for supplementation during ITT grafting for both its role as a vascularization enhancer and spermatogenesis regulator [77]. VEGF administration, either with subcutaneous injection directly to bovine testicular tissue grafting site [78] or during in vitro culture prior to germ cells transplantation [79], increased the number of seminiferous tubules containing elongating spermatids and germ cells survival, respectively. Moreover, in vitro culture of mouse SSCs with VEGF improved seminiferous tubules and vascularization reconstitution after ectopic allotransplantation of SSCs on the back skin of nude mice [77].

These encouraging results endorsed the use of nanoparticles containing VEGF as described below (see Section 2.10).

### 2.4. Using Protective Molecules to Reduce Ischemic Injury

Another strategy to limit damages to the testicular cells consists in actively protecting them from external insults during experimental manipulation. Hypoxic injury is associated with generation of reactive oxygen species, and different treatments have been tested trying to reduce oxidative stress.

*N*-acetylcysteine acts as an antioxidant regenerating the pool of intracellular glutathione, it protects the cell from oxidative stress caused by reactive oxygen species and reduces cell membrane lipid peroxidation [80]. Systemic administration of *N*-acetylcysteine has proven useful for germ cells protection during testicular torsion [81,82], and media supplementation with acetylcysteine during in vitro culture reduced germ cells apoptosis [83]. However, immersion for 5 minutes of a 1 mm³ fragment of frozen-thawed human ITT in a solution containing *N*-acetylcysteine before xenografting in nude mice associated with intraperitoneal administration of the same drug during the 5 days had no impact on spermatogonial survival, nor on seminiferous tubules cellular proliferation or apoptosis [31].

The impact of melatonin supplementation on the improvement of murine spermatogonial survival resulted in different outcomes. This drug has antioxidant and antiapoptotic properties that rely on the radical scavenger effect delivered by its indole derivate [84]. While melatonin appeared to be beneficial in SSC transplantation experiments [85,86], addition of melatonin to the vitrification-warming medium did not reduce expression of apoptotic genes in frozen/thawed murine ITT [87].

Further studies are therefore needed to investigate the impact of antioxidants molecules delivery on ITT, focusing on new molecules or on the way these drugs are administered.

Sustained and localized delivery systems might be useful to further improve the effects of already tested molecules (see Section 2.9).

### 2.5. Challenges to Achieve a Successful Transplantation of Human Immature Testicular Tissue

Because of an existing risk of zoonosis and epigenetic modification of genetic heritage, under no circumstances is xenotransplantation supposed to be considered as an option to produce sperm for future clinical use [88].

With regards to autotransplantation of cryoconserved ITT and before translation to clinical practice, the grafting technique needs to be improved with the objectives of increasing spermatogonial survival and achieving germ cells differentiation up to the haploid stage.

The main challenge lies in the absence of a useful model to study tissue transplantation, since the host environment of the mouse precludes proper interpretation of the outcomes.

However, efforts to mimic the physiologic endocrine environment of the peripubertal transition period and development of methods to accelerate angiogenesis and stabilization of the vascular connection between host and graft will probably represent the main challenges to explore.

The use of vascular growth factors in vivo is confronted with major hurdles, like their short half-life, the effects of distant distribution such as increased vascular permeability with risk of edema and hypotension [89,90], and the enhancement of tumor neo-angiogenesis [91,92]. These issues could be overcome by controlled release of biologically active substances confined to the site of interest [93] using encapsulation matrices and tailored nanoparticles. The latter approach also offers further options for testing local growth factors that act on the regulation of the transplanted spermatogonial niche.

### 2.6. Lessons Learned from Transplantation of Spermatogonial Stem Cells (SSCs)

An alternative option for fertility preservation and restoration is to produce and transplant functional germ stem cells into the patient once his cancer has been cured or gonadotoxic treatment has been completed.

Suspensions of isolated SSCs have been obtained from both animal [94] and human [95] tissue, offering the possibility of germ cell cryopreservation [96], as well as the perspective of fertility restoration through cell propagation and transplantation. According to the technique described by Brinster and Zimmerman, mouse ITT samples were incubated with collagenase and trypsin to digest the extracellular matrix, and the cell suspension subsequently isolated (at a cellular concentration ranging from 10^6^ to 10^7^ SSCs/mL) was micro-injected directly in the recipient mice seminiferous tubules [94]. The injection site has been the object of further investigations that pointed to ultrasound-guided injection in the rete testis as the best technique for SSCs transplantation to testicles of a size bigger than rodents’, like humans’ [97,98].

Mouse SSCs proved to be able to colonize the testicle stem cell niche after infusion into the seminiferous tubules of allogenic mice sterilized by means of busulfan exposure, further allowing spermatogenesis and progeny conception through natural mating [94]. Interestingly, xenotransplantation in nude mice testicles of rat [99] and hamster [100] testicular suspensions produced mature spermatozoa, although with structural abnormalities [101]. On the contrary, human adult [102] and prepubertal [103,104] testicular suspensions could only colonize the seminiferous tubules.

The overall encouraging results of SSC transplantation in animals endorsed an experimental setting involving human adult testicular tissue. Testicular cell suspensions of patients affected by non-Hodgkin lymphoma were obtained before chemotherapy, cryopreserved and retransplanted after cancer treatment, but unfortunately no follow-up information on the outcomes of this clinical trial has been reported [105,106].

### 2.7. Challenges to Achieve a Successful Transplantation of Human Immature SSCs

Several issues such as the isolation of a sufficient SSC number in the suspension, the lack of a complete knowledge of gonadotoxic treatment effects on the endocrine and paracrine environment of the patient’s SSC niche, and the possible cancer cell contamination of the cell suspension are still under investigation.

For the procedure to be efficient in colonizing the recipient seminiferous tubules, cell suspension should have a sufficient concentration of SSC. While SSCs represent only a small proportion of testicular cells [107], less than 10% of mice transplanted spermatogonial stem cells can form colonies in the recipient seminiferous tubules [108], and this percentage is presumed to also be low in human testis [109].

Using mouse testicular-derived cell suspension, increased cell concentrations from 10^6^ up to 10^7^ and 10^8^ cells/mL led to an increase in the colonization efficiency after transplantation to sterilized mice [108], while studies using human adult testicular derived cell suspensions met slightly different results. Suspensions obtained from human adult testicular tissue with different cells concentrations were compared between each other in two experiments. Cells concentrations of 49.7 × 10^6^ cells/mL and 51.5 × 10^6^ cells/mL, when compared respectively to 23.7 × 10^6^ cells/mL and 27.4 × 10^6^ cells/mL, did not improve the outcome of SSCs xenotransplantation to nude mice sterilized by Busulfan exposure [102,110]. Interestingly, in the second experiment, increasing the cell concentration from 10.3 × 10^6^ to 27.4 × 10^6^ cells/mL improved colonization of seminiferous tubules [110]. Therefore, SSC expansion would probably be necessary for clinical application, as the cellular concentration of the transplanted suspension influences the colonization efficiency.

Indeed, in vitro culture of SSC retrieved from cell suspensions could be used to obtain a sufficient spermatogonial number for a clinical application of SSC transplantation [104].

Enrichment of cell suspensions in SSCs is also possible using several methods, such as cell sorting followed by in vitro expansion [111] or differential plating [112]. However, these techniques still need to be refined because of partial results in terms of cell purity [113] and viability.

Besides germ cells depletion, testicular somatic cells may also be affected by gonadotoxic therapies. Indeed, direct damage of Sertoli cells has been demonstrated [114], and post-chemotherapy increased LH levels with reduced testosterone blood concentrations observed after cytotoxic therapies have been ascribed to Leydig cells’ impairments [115].

The possibility of cancer cells contamination of the cellular suspension would forbid any attempt of transplantation. To overcome this issue, both cell sorting and in vitro culture techniques have been developed. While encouraging results were obtained using cell sorting for animal testicular tissue [116], previous attempts using multi-parameter cell sorting strategies for human SSCs were only partially successful [117,118,119].

An overview of previous studies attempting to separate cancer cells from SSC suspension is provided in Table 2.

Hence these techniques need to be improved, although there exist some limiting factors. Indeed, on one hand, a potential similarity between antigens expressed on the membrane of human SSCs and leukemia cells might interfere with the sorting techniques, and on the other hand, no phenotypic marker for SSCs can differentiate them from other spermatogonial cell populations [3]. Laborious immune-phenotyping analysis of the malignant cells would therefore be necessary to define individual surface markers suitable for negative selection [117].

Another strategy proposed by Sadri-Ardekani et al. involved the use of a specific culture system that was able to successfully eliminate contaminating leukemic cells when co-cultured together with male germ cells. However, cells from different types of tumours could behave in different manners once exposed to specific culture conditions; hence, the same technique might not be applicable in every circumstance [122]. Furthermore, dissimilar cellular behaviors between the co-culture model and the scenario where a tissue invaded by malignant cells is dissociated might be expected. Another important concern is the potential epigenetic modification of cultured germ cells. While long term cell culture did not appear to affect the genetic heritage of mouse SSCs [123], human SSCs presented modifications in DNA-methylation after 50 days of in vitro culture [124]. Further studies are thus needed to explore the procedure and its consequences.

### 2.8. Lessons Learned from Reports on Cellular and Tissue Encapsulation and Perspectives Using Scaffolds

The limitations and flaws of ITT and SSC transplantation techniques that we itemized in the first part of this paper could be overcome by interventions on cellular support and on integration of donor grafts to the host. Such objectives have been explored in several domains besides the field of fertility preservation and belong to bioengineering.

Tissue engineering is an interdisciplinary field that combines principles from chemistry, materials engineering, and life sciences [125]. It is based on the association of a scaffold, cells, and bioactive molecules [126]. It aims to support cell viability and functionality, and at the same time it provides controlled and sustained delivery of single or multiple biological active molecules, such as growth factors and therapeutic drugs [127]. Bioactive cues can be either incorporated in the construct as free molecules, as nanomedicines, or both, depending on the desired release profiles and, ultimately, on the expected effects. Such composites can reestablish, maintain, or improve the condition of tissues or cells by reproducing the architecture and biochemical characteristics of the original organ or tissue [128]. Tissue engineering could lead to multiple strategies to improve ITT and SSC transplantation, to develop a proper study model or to elaborate a transplantable artificial testis.

#### 2.8.1. Cells or Tissues Encapsulation

Cells and tissue encapsulation in a three-dimensional environment using synthetic or biologically-derived matrices mainly aims to provide an environment that mimics the extracellular matrix (ECM) [129,130]. ECM is the non-cellular component that participates in the constitution of tissues and organs. It provides essential physical support for cells [131], allows cells communication and migration [132], and facilitates diffusion of cell nutrients and released products [133], and is thus required for tissue homeostasis. Testicular ECM plays a pivotal role in spermatogenesis through proteins like laminin and collagens that rule cellular interactions and thus differentiation of germ cells [134]. Modifications of this peculiar structure have indeed been observed when the normal function of the testis is compromised as in several pathologies associated with infertility [135].

Choosing the most appropriate material as an encapsulation matrix is essential for the outcome of tissue engineering constructs, and the designated components must satisfy many requirements, i.e., biocompatibility, biodegradability, and mechanical properties similar to those of the native tissue [131]. To be biocompatible, biomaterials must coexist and interact with the biological environment of the recipient without eliciting an excessive immune reaction [136] and eventually allowing a successful host integration [137]. Scaffold degradation products should also be non-toxic and should be eliminated from the body without interference with other organs [126,138].

Characteristics like stiffness and elasticity are influenced by the procedures used for matrix production, like the concentration of a given biomaterial, the degree of humidity, and temperature during preparation [139], or by the conditions to which the scaffold is subjected once implanted. For instance, an increase of temperature and hydration can reduce compressive modulus and compressive strength of poly(lactic-co-glycolic acid) (PLGA) scaffolds [140]. Furthermore, the material is supposed to allow artificial modifications of its physico-chemical characteristics in order to enhance specific cell behaviors and favor easy surgical manipulation of the graft [141]. An overview of characteristics to consider when a material is chosen and processed to be used for encapsulation is provided in Table 3.

#### 2.8.2. Use of Scaffolds

Tissue engineering provides an option for the reproduction of the structure of a tissue or organ. It consists in using a synthetic composite that could eventually be seeded and repopulated with isolated cells, providing an effective scaffold [48].

An alternative option to provide a scaffold for cellular support relies on the decellularization of tissues in order to obtain acellular matrices. It is a strategy that could be summarized in removing cells while preserving biological activity, biochemical composition, and three-dimensional structure of the ECM [144].

### 2.9. Bioactive Molecules Supplementation Using Nanoparticles

Entrapment of active molecules in nanoparticles is an approach used to circumvent unsuitable bioavailability, inadequate stability, and secondary effects at distant sites that may be observed with conventional systemic administration systems like tablets, capsules, and solutions. Nanomedicines incorporation in scaffolds would provide sustained drug delivery directly to the target site, and the molecule bio-distribution would no longer be related only to the drug itself, but also to carrier physicochemical properties [145].

Nanoparticles are solid colloidal particles with a diameter below 1 μm, in which the drug is either confined within a cavity enveloped by a membrane composed of a polymer, or it is dissolved within the polymer matrix [146].

The strategies that are employed to enhance the binding between a drug and the polymer composing the nanoparticle are both physical (charge interaction) and chemical (covalent binding) and depend on both the molecule to deliver and the carrier [147]. For instance, Huang et al. developed an encapsulation method for VEGF that relies on its ability to bind heparin. VEGF interacts with dextran sulfate via its heparin binding site and the polyelectrolyte complexes are stabilized by coacervation with chitosan [148].

Different molecules can be encapsulated or co-encapsulated in nanoparticles that can be combined in an implant, providing simultaneous [149] or sequential [150] drug administration according to the pharmacokinetic characteristics required for the specific therapeutic purpose.

Enhancing angiogenesis through vascular growth factor delivery is one the most frequent application of nanomedicine, and the possibility of obtaining a sequential delivery of multiple growth factors might help the reproduction of the process of angiogenesis.

Simultaneous release of VEGF and PDGF incorporated into fibrin scaffolds boosted angiogenesis in pancreatic islets grafts [151]. Nanoparticles of PLGA and poly(l-lactide) (PLLA) allowed simultaneous release of VEGF and FGF and sequential release of PDGF. This system has effectively enhanced vascularization in the rat aortic ring assay [150].

Drug release rates can be orchestrated, as material degradation can be programmed using either different molecular weights of the same polymer, different polymer formulation, or multiple drugs encapsulation within the same matrix but with different interaction mechanisms between the drug and the material [152].

### 2.10. Tissue-Engineering Applications for Testicular Tissue Transplantation

Encapsulation and local drug delivery systems have been the object of several reports concerning the testicle [129,141,153,154,155].

In a study addressing support matrices to optimize orthotopic avascular ITT auto-grafting in mice, Poels et al. evaluated two different hydrogels, one made of 1% alginate and another made of fibrin (30 mg/mL fibrinogen/30 IU/mL thrombin). Results showed a two-fold improvement in the survival of the subpopulation of spermatogonia not committed to differentiation (including the SSCs) with the use of the alginate matrix compared to fibrin gel (*p* < 0.05) [141]. This could be explained by differences in the structure of the materials that may influence the diffusion of nutrients and the invasion of vascular cells [156]. Indeed, the alginate hydrogel used presented a honeycomb structure with pores of 200 μm diameter, while the fibrin hydrogel had a nano-fibrous network with 1 μm pores [141]. Another reason for the increase in spermatogonial cell survival could be the intrinsic antioxidant properties of the oligo- and polysaccharides originating from algae such as alginate [157]. In the only previous experiment concerning human testicular tissue, encapsulation with alginate of testicular cells dissociated from seminiferous tubules of adult azoospermic patients with maturation arrest led to maturation of differentiated haploid germ cells during in vitro culture [153].

Alginate hydrogel displayed low cytotoxicity in 3D culture of mice prepubertal male germ cells [155]. Moreover, when used for encapsulation of bull germ cells during in vitro culture, it allowed differentiation up to the stage of haploid cells [129].

Such results suggest that alginate is an ideal candidate for tissue engineering of the testicle.

The effects of VEGF-loaded nanoparticles have been explored in an experiment involving orthotopic auto-graft of fresh mouse ITT. Use of dextran/chitosan nanoparticles delivering VEGF led to an increased graft vascular density at 5 days. However, this result was not maintained at 21 days post-implantation, suggesting a lack of stabilization of the neovascularization [141].

Any action aimed at increasing, accelerating the formation, and stabilizing newly formed vessels might promote graft survival and function. It is thus an important target to further improve ITT transplantation technique using tissue engineering approaches.

### 2.11. Future Directions for Fertility Restoration in Boys Using Transplantation of Prepubertal Cells or Tissues

The many differences in the previous experimental settings, such as different xenografting sites, hormone environment of the host mice, and donors’ characteristics like age, preexisting medical condition, concomitant gonadotoxic treatments, and donors’ unknown fertility potential, make the results of these reports somehow difficult to compare. In addition, a major limitation of studies on fertility preservation in prepubertal patients is the limited availability of human ITT.

However, the development of models, relying on the use of nanotechnology, on bioengineering, and on organoïds, provides further perspectives to the field.

New drug delivery strategies also open a vast window of opportunities, like the evaluation of new molecules for vascularization enhancement, the prevention of oxidative stress, and hormonal environment modulation, which would directly improve ITT and SSCs transplantation outcome.

Other ways to support gonadal cells or tissue in vivo might also be taken into consideration, like cell therapy. For example, locally injected allogenic mesenchymal stem cells were shown to improve spermatogonial survival after testicular torsion-induced hypoxia-reoxygenation in the rat [158].

The heterogeneous behavior of the different testicular cells populations when exposed to stress in vivo and in experimental conditions produces different responses and is yet to be fully investigated. In rats injected with ethanol in order to reproduce a model of stress, germ cells apoptosis was found to be enhanced, while Sertoli cells could activate pathways such as autophagy and mitophagy [159,160]. These pro-survival mechanisms might have implications that should be considered in situations when germ cells are exposed to important stress (such as during transplantation).

Germ cells could be seeded after in vitro and in vivo maturation [20] in testicular tissue decellularized matrix [161,162] to produce organoids, relying on the ability of mammalian cells to reproduce multi-cellular structures outside the body [163]. The knowledge resulting from these studies will certainly play a major role in the studies focalizing on the physiology of the testicle and could offer alternative models to the cell xenografting assay.

The creation of an artificial testicle would involve isolating human male germ cells from cryostored ITT or testicular cell suspensions, manipulating them safely and effectively in vitro and incorporating them in scaffolds possibly loaded with specific bioactive molecules. An eventual transplantation of this construct directly to the cancer cured infertile patient would be an ultimate goal of tissue engineering application to prepubertal male fertility preservation. A key issue in this process would be the repopulation of the scaffold, a process that could be enhanced by use of encapsulation with materials like alginate or collagen. Collagen already proved itself useful, as testicular cells isolated from juvenile rats and cultured in vitro in collagen sponges could form clusters composed of Sertoli cells, peritubular cells, and undifferentiated spermatogonia [154]. Scaffold obtained from cadaveric adult human testicles showed an effective decellularization and a preserved 3-D structure [161]. However, such tissue availability is limited, and its supply is rather complicated. Therefore, using animal-derived tissue would probably be a better option. With a view to restore the fertility of young boys exposed to cytotoxic treatments, the differences in physical and 3D properties between prepubertal and mature testicular tissue should be kept in mind. The perspective of using decellularized scaffold obtained from multi-transgenic swine-derived testicle might be an option that would help circumvent complications linked to the transplantation of scaffolds from animal origin. Tissues derived from such animals have already been authorized for clinical trials (e.g., for swine-derived pancreatic islets transplantation to diabetic human patients) [164], or have even entered clinical practice (e.g., for cardiac valve replacement) [165], and both the safety and feasibility of this procedures have been proven.

Further perspectives using the cell transplantation assay may involve the use of suspensions containing germ cells obtained from pluripotent stem cells, a cellular population that can be induced through a dedifferentiation process of somatic adult cells [166]. Mouse pluripotent stem cells could successfully differentiate up to the stage of spermatogonial-like cells and colonize seminiferous tubules once injected into adult testicles. Albeit with alterations of DNA methylation, this cellular population led to spermatogenesis and generation of fertile progeny [167]. Similar encouraging results have been obtained when mice primordial germ cells (retrieved from mice embryos) [168], and primordial germ cell-like cells (obtained from in vitro culture of induced pluripotent stem cells) [169] were transplanted to neonatal testicles. Human pluripotent stem cells successfully differentiated in vitro to primordial germ cell-like cells [170], although the potential of these cells for generation of more mature germ line cells is yet to be explored. So far, the most relevant benefit of this approach lies in its use as a disease model to study fertility restoration possibilities in cases of genetic abnormalites responsible for infertility [171].

## 3. Materials and Methods

We conducted a search on PubMed database (PubMED, National Center for Biotechnology Information, US National Institutes of Health, Bethesda, MD, USA) for the terms “Testicular Tissue transplantation” and “Spermatogonial stem cells transplantation”, representing the primary topic.

The studies linked to the principal subject of interest, published in English or French between 1984 and 15 December 2017 were referenced in this review. The bibliography of the cited study has been likewise reviewed and used as source for reports cited in the discussion.

**Figure 2:**
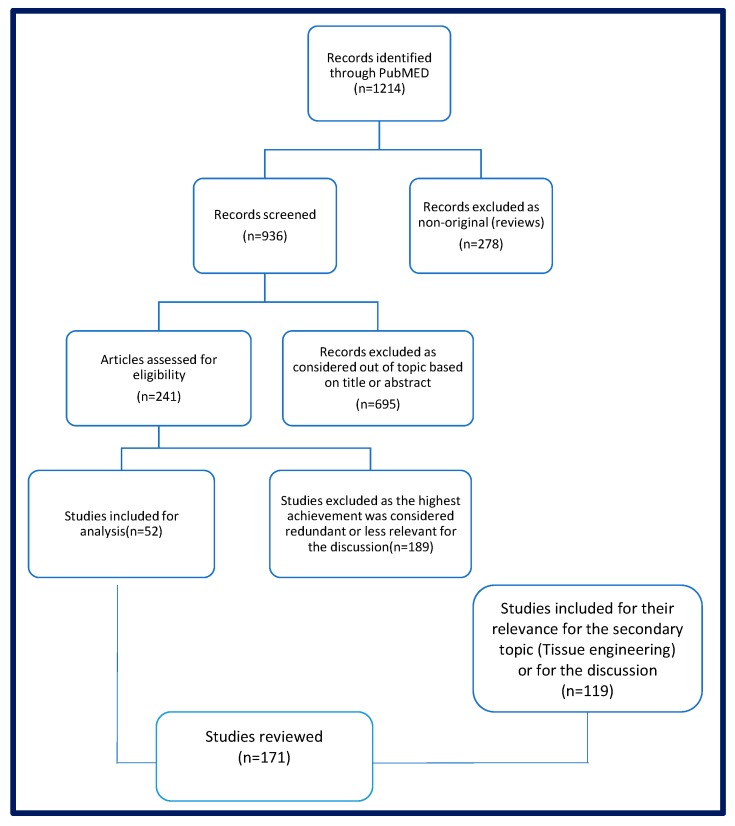
Flow chart that describes the articles selection process for inclusion in the review.

In Figure 2, a flow chart describes the article selection process.

## 4. Conclusions

The many perspectives we itemized could offer, alone or combined with each other, several hints for male prepubertal fertility restoration. Efforts have been made in the last decade for cryobanking of ITT, but a shift from an experimental to a clinical practice of these procedures needs further reassurance that one technique can become effective to restore a patient’s fertility. Insights in the processes that rule spermatogenesis would help to clarify the reproductive potential of spermatogonia that survive after prepubertal tissue cryopreservation. Distinction between different populations of germ cells is mandatory to understand whether the germ cells precursors present in cryostored tissues can lead to fertility restoration. Indeed, in experimental conditions, a differentiation blockade at the diploid cell stage has always been encountered so far, while for the procedure to be considered effective, a differentiation up to the stage of haploid cells is necessary. Further studies are thus needed to explore the biology of spermatogenesis and to find out if the techniques that have already been effective with animal tissue could also be successful with human tissue. Cells sustainment and molecule supplementation could be options to explore.

In conclusion, tissue engineering might represent not only a progress in fertility restoration, but it could also offer a model to study prepubertal testicle physiology.

## Figures and Tables

**Table 1:** Reports on xenotransplantation of post- and pre-pubertal human testicular tissue in nude mice.

Reference	Donor	Grafting Site	Graft Size	Cryopreserved or Fresh	Castrated Host	Outcome
Wyns et al., 2007; [12]	Prepubertal	Peritoneum scrotal bursa	2–9 mm^3^	F/T	yes	SG survival after 3 weeks: 14 %
Wyns et al., 2008; [16]	Prepubertal	Peritoneum scrotal bursa	2–8 mm^3^	F/T	yes	SG survival 3.7%, numerous premeiotic spermatocytes, a few spermatocytes at the pachytene stage and spermatid and spermatozoa-like cells, without expression of the meiotic and post-meiotic markers
Goossens et al., 2008; [27]	Prepubertal 10 and 11 y.o.	Back skin	2–10 mm^3^	fresh	no	Some rare SG survival after 4–9 months
Sato et al., 2010; [28]	3 y.o. testicular hemangioma	Back skin	0.5–1 mm^3^	fresh	yes	Pachytene spermatocytes after one year
Van Saen et al., 2013; [29]	Prepubertal	Intra- testicular	1.5–3 mm^3^	Fresh and F/T	No	No effect of FSH administration or slow-freezing primary pachytene spermatocytes 9 and 12 months after grafting
Poels et al., 2013; [30]	Prepubertal	Peritoneum scrotal bursea	1 mm^3^	Fresh, slow-frozen and vitrified	yes	SG survival after 6 months: 3.4%, 4.1%, and 7.3%, respectively, for fresh, slow-frozen-thawed and vitrified-warmed tissue. No statistical significant difference between three groups.
Poels et al 2014; [31]	Prepubertal	Peritoneum scrotal bursa	1 mm^3^	F/T	yes	SG survival after 5 days, 67%, 63%, and 53%, respectively, for slow-frozen tissue, slow-frozen tissue supplemented with NAC, and slow-frozen tissue supplemented with FSH and testosterone. No impact of NAC or FSH/Testosterone supplementation on SG survival
Schlatt et al., 2006; [32]	Adult4 Azoospermic patients, 1 Cancer survivor, 1 Testicular cancer patient, 3 transsexual patients.	Back skin	0.5–1 mm^3^	fresh	yes	Occasional Type A SG survival No correction of spermatogenesis disruption after xenografting
Geens et al., 2006; [34]	Adult	Back skin	4 mm^3^	fresh	yes	SG survival 33% < 120 d 14% > 120 d
Van Saen et al., 2011; [35]	Prepubertal POST-CHEMOTHERAPY Postpubertal (12 and 13 y.o.)	Intra-testicular	6 mm^3^	F/T	no	4 months: SG survival 0,2/ST. 9 months: SG survival 0,6/ST. Higher SG survival in xenografts from the postpubertal donors. No SG differentiation in younger patients ‘tissue, two older donors’ tissue with differentiation up to primary spermatocyte and secondary spermatocytes in the oldest donor after 9 months.

SG: = spermatogonia; y.o.: years old; ST: seminiferous tubule; F/T: Frozen/Thawed; hCG: human Chorionic Gonadotropin; FSH: Follicle Stimulating Hormone; NAC: *N*-Acetyl-Cysteine.

**Table 2:** Segregation of cancerous cells from human and animal testicular tissue.

Reference	Species	Technique	Outcome (Residual Contamination/Contamination of Samples or Contamination of Mice after Transplantation)
Fujita et al., 2005; [116]	Mouse	FACS	No contamination of recipient mice
Fujita et al., 2006; [117]	Human	FACS	Malignant cells in 1/8 in vitro cultures
Geens et al., 2007; [118]	Mouse	MACS + FACS	Malignant cells in 1/32 in vitro cultures 43% of mice contaminated after transplantation
	Human	FACS	10/11 contaminated cultures
Dovey et al., 2013; [119]	Human	FACS	Post FACS Purity check was only 98.8–99.9% No tumour formation after xenotransplantation of sorted cell suspension to 55 nude mice (but tumour formation after contaminated cell transplantation was only 23–55%)
Hou et al., 2007; [120]	Rat	FACS	Germ cells selection or leukaemia cells isolation: contamination of 2/3 and 2/2 recipient rats Germ cell selection and leukaemia cells isolation: survival of all recipient rats
Hermann et al., 2011; [121]	Non-human primates	FACS	No tumour after nude mouse transplantation in 3 of 4 cell colonies
Sadri-Aderkani et al., 2014; [122]	Human	In vitro culture	Acute lymphoblastic leukaemia cells undetectable after 26 d

FACS: fluorescence activated cell sorting; MACS: magnetic activated cell sorting; d: days.

**Table 3:** Matrix mechanical characteristics that impact cell or tissue function.

Matrix Mechanical Characteristics that Impact Cell or Tissue Function
• Pore size and morphology (Chan et al., 2008); [142]
• Elasticity (Janson et al., 2015); [131]
• Stiffness (Xia et al., 2017); [143]
• Hydration degree (Wu et al., 2006); [140]

## Abbreviations

SSC	Spermatogonial stem cells
ITT	Immature testicular tissue
SG	Spermatogonia
ST	Seminiferous tubules
F/T	Frozen/Thawed
FSH	Follicle stimulating Hormone
LH	Luteinizing Hormone
HcG	Human chorionic Gonadotropin
VEGF	Vascular Endothelial Growth Factor
PDGF	Platelet Derived Growth Factor
FGF	Fibroblast Growth Factor
MACS	Magnetic Activated Cell Sorting
FACS	Fluorescence Activated Cell Sorting
